# Two New Differential Signs in Dislocations of the Shoulder

**Published:** 1875-07

**Authors:** Frank H. Hamilton

**Affiliations:** Being a portion of a Clinical Lecture delivered at Bellevue Hospital, after the presentation of a case of Subcoracoid Dislocation


### TWO NEW DIFFERENTIAL SIGNS IN DISLOCATIONS OF THE SHOULDER.

BY PROF. FRANK H. HAMILTON, M. D.

*Being a portion of a Clinical Lecture delivered at Bellevue Hospital, after the presentation of a case of Subcoracoid Dislocation.*

The examples of errors of diagnosis in the case of injuries involving the shoulder-joint are very frequent. My personal experience furnishes me with probably forty or fifty cases in which the head of the humerus has been supposed to be dislocated when it was not; or in which it has been supposed to be broken when it was not. For this reason it is important that you be informed of every known means of diagnosis; and to those which are already known and published I will now add two more, of which you will be able pretty often to avail yourselves.

When the head of the humerus is in its socket it projects outwards, beyond the extremity of the acromion process, from half an inch to an inch; varying more or less according to the age and size of the person. It projects also in front of the acromion process a little, but not at all behind.

In case of a dislocation, in whatever direction the head of the humerus is displaced, there can be no bony projection outwards beyond the acromion process. This fact may be ascertained always, unless there is very great swelling of the soft parts over the point of the shoulder; but it will be necessary that you should be familiar with the natural outline of the acromion process, and this is a study which medical students and medical men too much neglect, namely, the study of the natural form of the surface of the body, or what I call "Superficial Anatomy." You must learn to know where is the outer end of the clavicle, where is the outer end of the acromion process, and where is the coracoid process, if you expect to determine the existence or absence of a dislocation of the shoulder. This exercise you can pursue in your bed-rooms, on your own persons or on the persons of others. With a camel's hair pencil, moistened with the tincture of iodine, you can mark out upon the skin the line of the clavicle, acromion process, spine of the scapula, etc. In attempting this for the first time you will probably find that there is much to learn that you did not know before, however thoroughly you have studied the anatomy of the shoulder in the dissecting-room, when the skin is removed. The same applies to all the other joints of the body; and now you will understand why some men, perhaps wholly ignorant of anatomy as it is usually taught, but familiar by long practice with superficial anatomy, will recognize in a moment the nature of a joint injury, which you may fail after a careful examination to detect.

Let us return to the consideration of the two special signs of shoulder-joint dislocation (liable to only one exception, as I shall hereafter explain), which I wish to add to those already given by surgical writers.

***First.*** While the head of the humerus remains in its socket, if a rule be laid upon the outside of the arm from the shoulder to the elbow it will not touch the acromion process but will be distant from it at least half an inch, generally one inch or more. On the other hand, if the bone is removed from the socket in whatever direction it may be displaced, whether forwards, downwards, or backwards, unless the shoulder is much swollen, the rule, placed in the manner above stated, will touch the acromion process.

*Second.* If standing behind the patient (in case of the right shoulder) the thunb and forefinger of the left hand is made to grasp the top of the shoulder in such a manner as that the inter-digital commissure shall rest upon the acromion process, just outside of the acromio-clavicular articulation ; and if then the finger and thumb are dropped perpendicularly, the tip of the finger will (in case the head of the humerus is not dislocated) rest upon the centre of the round upper extremity of the humerus, as it projects in front of the acromion process, while the end of the thumb will rest upon the head of the humerus behind ; but the head will be felt indistinctly by the thumb, for the reason that, instead of projecting as it does in front, it actually recedes a little beneath the acromion process. Up to this moment the surgeon may entertain some dout whether he is actuallly grasping with his thumb and finger the head of the bone; but if he now moves the elbow of the injured limb forwards, so as to carry the head of the humerus backwards in its socket, he will feel it press strongly upon the thumb, and this will be conclusive. If a dislocation exists, the head of the bone cannot be felt in this situation, and by the thumb thus placed.

I have told you that both of these differential signs, in their application to shoulder-joints injuries, are liable to one exception.—The phenomena would be the same, so far as these two signs are concerned, whether there was a dislocation of the head of the humerus, or a fracture with displacement of the neck of the scapula. The latter accident must, therefore, be first excluded by a careful application of the rules of diagnossis given in our treatises upon surgery ; but that upon which you can most safely rely is the relative infrequency of the two accidents. It is doubtful whether a long and active surgical practice will ever furnish you with an example of fracture of the neck of the scapula, while you'll meet with a great many cases of dislocation of the shoulder.—*New York Medical Record.*

